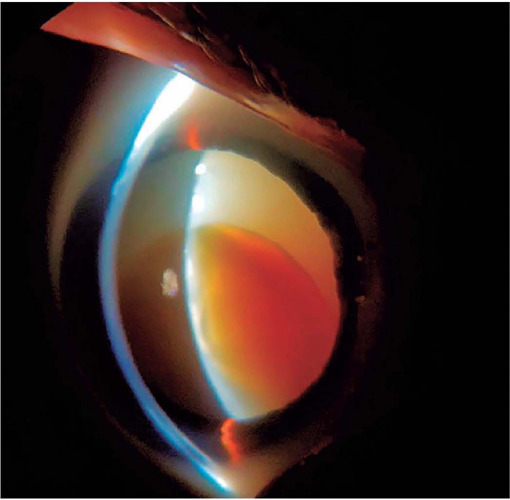# Morgagnian cataract

**DOI:** 10.5935/0004-2749.2024-0121

**Published:** 2024-06-20

**Authors:** Fabio Marinho, Nicole B. M. Almeida, Newton Kara-Junior

**Affiliations:** 1 Ophthalmology department, Hospital das Clínicas, Universidade de São Paulo, São Paulo, SP, Brazil

Morgagnian cataract, a special form of corticonuclear cataract^(^1^)^, is a hypermature lens in which the cortex liquifies and the dense nucleus sinks inferiorly due to gravity^(^2^)^. In this type of cataract, the process of fiber dissolution accelerates and occurs en masse^(^1^)^. A spontaneous rupture can occur, causing severe intraocular inflammation^(^3^)^; therefore, early treatment is required^(^3^)^. However, surgery for this type of cataract has a high incidence of complications^(^4^)^ that include rigid anterior lens capsule, weak zonules, milky cortex with inadequate support for rhexis, and hard nucleus, leading to the risk of posterior capsular rupture, zonulodialysis, vitreous loss, nucleus drop, or inadvertent removal of the entire capsular bag^(^1^)^.

**Figure:**